# Unforeseen nodal upstaging in patients undergoing segmentectomy without frozen section: a multicenter retrospective cohort study

**DOI:** 10.1007/s00464-025-11612-9

**Published:** 2025-02-13

**Authors:** Lin Huang, Alessandro Brunelli, Demetrios Stefanou, Edoardo Zanfrini, Abid Donlagic, Michel Gonzalez, René Horsleben Petersen

**Affiliations:** 1 Department of Cardiothoracic Surgery, Copenhagen University Hospital, Rigshospitalet, Blegdamsvej 9, 2100 Copenhagen Ø, Denmark; 2 Department of Thoracic Surgery, St. James’s University Hospital, Leeds, UK; 3 Department of Thoracic Surgery, University Hospital of Lausanne, Lausanne, Switzerland

**Keywords:** Segmentectomy, Frozen section, Non-small cell lung cancer, Nodal upstaging, Maximum standardized uptake values

## Abstract

**Objective:**

The study aimed to evaluate the risk of unforeseen nodal upstaging (pN+) after pulmonary segmentectomy without intraoperative frozen section.

**Methods:**

We conducted a retrospective analysis for consecutive patients who underwent segmentectomy for clinical stage IA1-2 non-small cell lung cancer (cIA1-2 NSCLC) in three centers between January 2017 and December 2022. A backward stepwise logistic regression analysis for variables with *P* < 0.1 in univariable analysis was performed to predict pN+. Kaplan–Meier analysis with log-rank test evaluated the discrepancy for overall (OS) and recurrence-free survivals (RFS).

**Results:**

Among 478 patients included in the final analysis, 19 (4.0%) had pN+, including 10 (2.1%) pN1, 6 (1.3%) pN2, and 3 (0.6%) pN1+2. With a median follow-up of 23.5 months (interquartile range 12.6–39.0), patients with pN+ had poorer OS compared to those with pN0 (3-year OS: 70.2% vs. 89.7%, *P* = 0.002). However, there was no significant difference in RFS and recurrence. The maximum standardized uptake value (SUVmax) of tumor in positron emission tomography scan ≥ 4.5 (versus < 4.5) was the only independent factor for pN + (odds ratio 3.5). Patients with a SUVmax ≥ 4.5 had 7.3% pN+, which was associated with poorer OS and similar RFS and recurrence compared to pN0. In contrast, those with a SUVmax < 4.5 had 2.2% pN+, which had comparable recurrence and survival to pN0.

**Conclusion:**

Unforeseen nodal upstaging in segmentectomy for cIA1-2 NSCLC is low. Frozen section of lymph nodes may be necessary for lesions with high metabolic activity.

**Supplementary Information:**

The online version contains supplementary material available at 10.1007/s00464-025-11612-9.

The latest NCCN® guidelines [1] recommend considering sublobar resection, preferably segmentectomy, for peripheral T1a-bN0 non-small cell lung cancer (cIA1-2 NSCLC ≤ 2 cm). Lobectomy is considered the standardized surgical treatment for NSCLC with pathologic nodal upstaging (pN +). Frozen section during segmentectomy may assist surgeons in deciding whether to convert to lobectomy. According to the recent consensus statement from the European Society of Thoracic Surgeons [2], 75% of the expert panel recommends performing frozen section on hilar lymph nodes during segmentectomy. However, this procedure has a sensitivity of only 85% and may potentially prolong surgical time and increase costs [3]. Hence, the variability of frozen section examination remains in clinical trials [4–6]. In addition, there is currently no procedure-specific data available for the incidence of unforeseen pN + in cIA1-2 NSCLC, making it difficult to further evaluate its impact.

Accordingly, this study aimed to evaluate risks of unforeseen pN + following segmentectomy in a consecutive cohort of patients with cIA1-2 NSCLC and operated in units where intraoperative frozen section of lymph nodes was not performed.

## Methods

The Research and Innovation Department of Leeds Teaching Hospitals, the Ethics Committee of the University Hospital of Lausanne (2024-00173), and the Regional Review Boards of Denmark (R-22063267) approved this study. The informed consent was waived.

### Study design and patient population

We conducted a retrospective analysis of prospectively collected data from consecutive segmentectomies for cIA1-2 NSCLC, between January 2017 and December 2022, at three European university hospitals.

The primary outcome was the incidence of unexpected pN + at definitive pathology, while secondary outcomes included identifying predictors for pN+, as well as analyzing recurrence, overall survival (OS), and recurrence-free survival (RFS). Locoregional recurrence was defined as tumor relapse in ipsilateral preserved lobe, hilar or mediastinal lymph node. Other relapses were classified as distant recurrence. The OS was calculated in months from the day of surgery until the day of death or last follow-up, while the RFS was calculated in months from the day of surgery until the day of any recurrence, death or last follow-up.

Additional variables included age, body mass index, gender, lung function, comorbidities, Eastern Cooperative Oncology Group performance score, clinical and pathologic T stage (according to the 8th edition of the TNM classification system), maximum standardized uptake values (SUVmax, classified into two groups based on a C-index statistical analysis for unforeseen pN+, which established 4.5 as the cut-off value), consolidation tumor ratio (C/T ratio), location of lesions, surgical access, type of segmentectomy, status of lymph node dissection, histology of tumor (according to the World Health Organization Classification), tumor size, margin distance, residual tumor, pleural invasion, length of stay, complications, adjuvant therapy, and completion lobectomy.

The results of this study were reported in compliance with the Strengthening the Reporting of Observational Studies in Epidemiology (STROBE) guidelines [7].

### Procedures

Preoperative evaluation for patients included thin-section computed tomography (CT) and positron emission tomography (PET) scans. In cases where lymph node involvement was suspected radiologically, endobronchial ultrasound or mediastinoscopy was conducted. A transthoracic CT-guided biopsy or bronchoscopic biopsy of the lesion was performed to determine histology if no technical difficulties were encountered. Frozen section of lymph nodes was not performed in any of the 3 centers during the study period. The choice of minimally invasive surgery or thoracotomy depended on the surgeon’s preference. Additionally, each center employs similar surgical techniques, including systematic lymph node dissection, margin definitions, and the selective use of indocyanine green fluorescence or three-dimensional reconstruction. The follow-up programs included CT examinations every three months for the first two years and biannual CT for the subsequent three years. Therapeutic strategies for all cases were individually discussed in a multidisciplinary tumor board.

### Statistical analysis

The Kolmogorov–Smirnov and Shapiro–Wilk tests confirmed the abnormal distribution of all continuous variables. These were presented as medians with interquartile ranges (IQR), and categorical variables were presented as counts and proportions. Missing data were methodically managed through multiple imputations. We applied chained equations to create 20 imputed datasets, which were then analyzed using multiple regression models. Pooled estimates were subsequently obtained following Rubin’s rule. Univariable analysis was conducted by the Mann–Whitney *U* test for continuous variables and Fisher’s exact test (if > 20% of expected cell counts less than 5) or Pearson's chi-squared test for categorical variables. We employed backward stepwise logistic regression analysis for preoperative variables with a P < 0.1 in univariable analysis to predict unforeseen pN+. Kaplan–Meier analysis with the log-rank test was utilized to assess OS and RFS. Subgroup analysis based on predictors was performed for primary and secondary outcomes. Statistical significance was set at *P* < 0.05. All analyses were conducted using R Software (version 4.4.1, R Foundation for Statistical Computing, Vienna, Austria).

## Results

Of the 478 eligible patients, 19 (4.0%) had unforeseen pN + at final pathology. This included 10 patients (2.1%) with pN1, distributed as follows: two at station 10, five at station 11, two at station 12, and one at station 13. Additionally, there were 6 patients (1.3%) with pN2, with two at station 2R, one at station 5, and 3 at station 7. Furthermore, three patients (0.6%) had pN1 + 2: one involving stations 4L and 10, one involving the stations 5 and 11, and one involving stations 5 and 12. The details across the participating hospitals are shown in Supplementary Table 1.

### Demographics and preoperative characteristics

In the overall cohort, the median age was 69 years (IQR 63–75), with 43.1% males and 56.9% females. Tumors were classified as 35.1% cT1a and 64.9% cT1b. Additionally, 79.5% of tumors were in the outer one-third of the parenchyma and 69.0% had a C/T ratio > 0.5. The median SUVmax was 3.0 (IQR 1.9–5.8) with 163 patients in the hypermetabolic group and 315 in the hypometabolic group. Patients with pN + did not exhibit statistically significant differences in preoperative variables compared to those with pN0 (Table 1).

**Table 1:** Demographics, preoperative, and intraoperative clinical characteristics

Variables^a^	Total (*n* = 478)	pN0 (*n* = 459)	pN + (*n* = 19)	*P* value
Age, year	69 (63–75)	69 (63–75)	69 (59–71)	0.362
BMI, kg/m^2^	25.8 (22.7–28.6)	25.8 (23.0–28.4)	25.0 (20.7–30.9)	0.924
Gender				0.351
Male	206 (43.1%)	200 (43.6%)	6 (31.6%)	
Female	272 (56.9%)	259 (56.4%)	13 (68.4%)	
FEV_1_, %pre	86 (69–100)	86 (69–100)	84 (67–102)	0.904
DLCO, %pre	70 (60–85)	70 (60–84)	80 (63–96)	0.189
CAD	86 (18.0%)	83 (18.1%)	3 (15.8%)	1.000
CVD	52 (10.9%)	49 (10.7%)	3 (15.8%)	0.449
CCI, score	3 (1–5)	3 (1–5)	2 (0–5)	0.305
ECOG performance score				0.667
0	290 (60.7%)	277 (60.3%)	13 (68.4%)	
1	155 (32.4%)	149 (32.5%)	6 (31.6%)	
2	28 (5.9%)	28 (6.1%)	0 (0.0%)	
3	5 (1.0%)	5 (1.1%)	0 (0.0%)	
Clinical T stage				0.227
T1a	168 (35.1%)	164 (35.7%)	4 (21.1%)	
T1b	310 (64.9%)	295 (64.3%)	15 (78.9%)	
PET/CT SUVmax	3.0 (1.9–5.8)	2.9 (1.9–5.6)	5.3 (2.0–7.0)	0.089
≥ 4.5	163 (34.1%)	151 (32.9%)	12 (63.2%)	
< 4.5	315 (65.9%)	308 (67.1%)	7 (36.8%)	
C/T ratio				0.098
GGO	270 (56.5%)	263 (57.3%)	7 (36.8%)	
0	52 (10.9%)	51 (11.1%)	1 (5.3%)	
≤ 0.5	96 (20.1%)	94 (20.5%)	2 (10.5%)	
> 0.5	122 (25.5%)	118 (25.7%)	4 (21.1%)	
Solid (1)	208 (43.5%)	196 (42.7%)	12 (63.2%)	
Peripheral location	380 (79.5%)	366 (79.7%)	14 (73.7%)	0.561
Lobar location of lesion				0.940
Upper lobe	278 (58.2%)	266 (58.0%)	12 (63.2%)	
Middle lobe	1 (0.2%)	1 (0.2%)	0 (0.0%)	
Lower lobe	194 (40.6%)	187 (40.7%)	7 (36.8%)	
Bi-lobe^*^	5 (1.0%)	5 (1.1%)	0 (0.0%)	
Side of thorax for lesion				1.000
Left side	241 (50.4%)	231 (50.3%)	10 (52.6%)	
Right side	237 (49.6%)	228 (49.7%)	9 (47.4%)	
Surgical access				0.882
Open	1 (0.2%)	1 (0.2%)	0 (0.0%)	
VATS	472 (98.7%)	453 (98.7%)	19 (100.0%)	
RATS	5 (1.0%)	5 (1.1%)	0 (0.0%)	
Conversion to open	8/477 (1.7%)	8/458 (1.7%)	0/19 (0.0%)	1.000
Complex segmentectomy	241 (50.4%)	232 (50.5%)	9 (47.4%)	0.819
Number of segments resected	1 (1–2)	1 (1–2)	1 (1–2)	0.949
Single	292 (61.1%)	280 (61.0%)	12 (63.2%)	
Multiple	186 (38.9%)	179 (39.0%)	7 (36.8%)	
Total lymph nodes dissected	6 (4–10)	6 (4–10)	7 (4–10)	0.797
N1 stations dissected	1 (1–2)	1 (1–2)	2 (1–2)	0.177
N2 stations dissected	2 (2–3)	2 (2–3)	2 (2–3)	0.876

BMI, body mass index; CAD, coronary artery disease; CCI, Charlson Comorbidity index; CVD, cerebrovascular disease; C/T ratio, consolidation tumor ratio; DLCO, diffusing capacity of carbon monoxide; ECOG, Eastern Cooperative Oncology Group; FEV_1_, forced expiratory volume in 1 s; GGO, ground-glass opacity; SUVmax, maximum standardized uptake values; RATS, robotic-assisted thoracoscopic surgery; VATS, video-assisted thoracoscopic surgery

*Bi-lobe included one middle lobe + lower lobe and four upper lobe + lower lobe

^a^Continuous variables were presented as median (interquartile range) and categorical variables were presented as number (proportion)

### Intraoperative characteristics

Nearly, all the patients in the study (99.7%) underwent minimally invasive surgery. The superior segment (S6) resection (27.4%) was the most frequent. Complex segmentectomies accounted for 50.4% of the procedures. Moreover, 61.1% of the patients had a single segment removed, while 38.9% had multiple segments removed. The median total number of lymph nodes dissected was 6 (IQR 4–10), with a median of 1 N1 station dissected (IQR 1–2) and a median of 2 N2 stations dissected (IQR 2–3). There were no significant differences in intraoperative characteristics identified when comparing patients with pN0 to those with pN+ (Tables 1, 2).

**Table 2:** Segments resected

Segments^a^	Total (*n* = 478)	pN0 (*n* = 459)	pN + (*n* = 19)	*P* value
S1	71 (14.9%)	68 (14.8%)	3 (15.8%)	0.949
S1 + 2	37 (7.7%)	36 (7.8%)	1 (5.3%)	
S1 + 2 + 3	58 (12.1%)	54 (11.8%)	4 (21.1%)	
S1 + 3	2 (0.4%)	2 (0.4%)	0 (0.0%)	
S1 + 8	1 (0.2%)	1 (0.2%)	0 (0.0%)	
S2	50 (10.5%)	48 (10.5%)	2 (10.5%)	
S2 + 4 + 5	2 (0.4%)	2 (0.4%)	0 (0.0%)	
S2 + 6	2 (0.4%)	2 (0.4%)	0 (0.0%)	
S2 + 10	1 (0.2%)	1 (0.2%)	0 (0.0%)	
S3	15 (3.1%)	14 (3.1%)	1 (5.3%)	
S3 + 4 + 5	3 (0.6%)	3 (0.7%)	0 (0.0%)	
S4	1 (0.2%)	1 (0.2%)	0 (0.0%)	
S4 + 5	34 (7.1%)	33 (7.2%)	1 (5.3%)	
S5	1 (0.2%)	1 (0.2%)	0 (0.0%)	
S5 + 6	1 (0.2%)	1 (0.2%)	0 (0.0%)	
S6	131 (27.4%)	126 (27.5%)	5 (26.3%)	
S6 + 9	2 (0.4%)	2 (0.4%)	0 (0.0%)	
S6 + 10	2 (0.4%)	1 (0.2%)	1 (5.3%)	
S7	1 (0.2%)	1 (0.2%)	0 (0.0%)	
S7 + 8	9 (1.9%)	9 (2.0%)	0 (0.0%)	
S7 + 8 + 9	4 (0.8%)	4 (0.9%)	0 (0.0%)	
S7 + 8 + 9 + 10	10 (2.1%)	10 (2.2%)	0 (0.0%)	
S8	13 (2.7%)	12 (2.6%)	1 (5.3%)	
S8 + 9	2 (0.4%)	2 (0.4%)	0 (0.0%)	
S8 + 9 + 10	3 (0.6%)	3 (0.7%)	0 (0.0%)	
S9	1 (0.2%)	1 (0.2%)	0 (0.0%)	
S9 + 10	13 (2.7%)	13 (2.8%)	0 (0.0%)	
S10	8 (1.7%)	8 (1.7%)	0 (0.0%)	

^a^Categorical variables were presented as number (proportion)

### Pathologic and postoperative characteristics

Adenocarcinoma (83.7%) was the predominant histologic type. The median tumor size was 14 mm (IQR 10–18). The median margin distance measured was 15 (IQR 6–25) mm, with a microscopic positive margin rate (R1) of 0.6%. Pleural invasion was presented in 9.6% of cases. Comparing patients with pN0 to those with pN+, the only significant difference observed was in the histologic type of NSCLC. Regarding postoperative outcomes, the cohort had a median length of stay of 4 days (IQR 3–6) with 17.4% experiencing cardiopulmonary complications. Additionally, 7.1% of patients received adjuvant therapy and 0.8% underwent completion lobectomy. No statistical differences were observed between the two groups, except that a higher proportion of patients in the pN + group received adjuvant systemic therapy compared to those in the pN0 group (*P* < 0.001). (Table 3).

**Table 3:** Pathologic and postoperative outcomes

Variables^a^	Total (*n* = 478)	pN0 (*n* = 459)	pN + (*n* = 19)	*P* value
Histology				**< 0.001**
Adenocarcinoma in situ	25 (5.2%)	25 (5.4%)	0 (0.0%)	
Minimally invasive adenocarcinoma	6 (1.3%)	6 (1.3%)	0 (0.0%)	
Invasive adenocarcinoma	369 (77.2%)	353 (76.9%)	16 (84.2%)	
Squamous cell carcinoma	67 (14.0%)	66 (14.4%)	1 (5.3%)	
Adenosquamous cell carcinoma	4 (0.8%)	2 (0.4%)	2 (10.5%)	
Large cell carcinoma	5 (1.0%)	5 (1.1%)	0 (0.0%)	
Mixed of squamous cell and large cell carcinoma	2 (0.4%)	2 (0.4%)	0 (0.0%)	
Tumor size, mm	14 (10–18)	13 (10–18)	15 (12–20)	
Pathologic T stage				0.665
Tis	25 (5.2%)	25 (5.4%)	0 (0.0%)	
T1a (min)	6 (1.3%)	6 (1.3%)	0 (0.0%)	
T1a	125 (26.2%)	122 (26.6%)	3 (15.8%)	
T1b	230 (48.1%)	218 (47.5%)	12 (63.2%)	
T1c	34 (7.1%)	33 (7.2%)	1 (5.3%)	
T2a	49 (10.3%)	46 (10.0%)	3 (15.8%)	
T3	9 (1.9%)	9 (2.0%)	0 (0.0%)	
Margin distance, mm	15 (6–25)	15 (6–25)	14 (5–20)	
Residual tumor				0.115
R0	475 (99.4%)	457 (99.6%)	19 (94.7%)	
R1	3 (0.6%)	2 (0.4%)	1 (5.3%)	
Pleural invasion	46 (9.6%)	43 (9.4%)	3 (15.8%)	0.413
Length of stay, day	4 (3–6)	4 (3–6)	3 (2–5)	0.185
Cardiopulmonary complications	83 (17.4%)	78 (17.0%)	5 (26.3%)	0.348
Adjuvant therapy	34 (7.1%)	20 (4.4%)	14 (73.7%)	**< 0.001**
Completion lobectomy	4 (0.8%)	4 (0.9%)	0 (0.0%)	1.000
Recurrence				
Any	49 (10.3%)	46 (10.0%)	3 (15.8%)	0.430
Locoregional	26 (5.5%)	25 (5.4%)	1 (5.3%)	1.000
Distant	23 (4.8%)	21 (4.6%)	2 (10.5%)	0.231

Bolded *p*-values indicate statistical significance

^a^Continuous variables were presented as median (interquartile range) and categorical variables were presented as number (proportion)

### Predictors for unforeseen pN + 

In univariable analysis, the C/T ratio (< 1 vs. = 1) and SUVmax (≥ 4.5 vs. < 4.5) were the only two variables selected for inclusion in the multivariable analysis. Multivariable stepwise logistic regression analysis revealed that a SUVmax ≥ 4.5 was the sole-independent risk factor for pN + (Table 4).

**Table 4:** Results of the stepwise logistic regression analysis

Variable	Coefficient	SE	OR	95% CI	*P* value
SUVmax ≥ 4.5 (reference: < 4.5)	1.25	0.49	3.50	1.35–9.06	0.010

OR, odds ratio; SUVmax, maximum standardized uptake values; SE, standard error; 95% CI, 95% confidence interval

### Recurrence rate and survivals

**Fig. 1:**
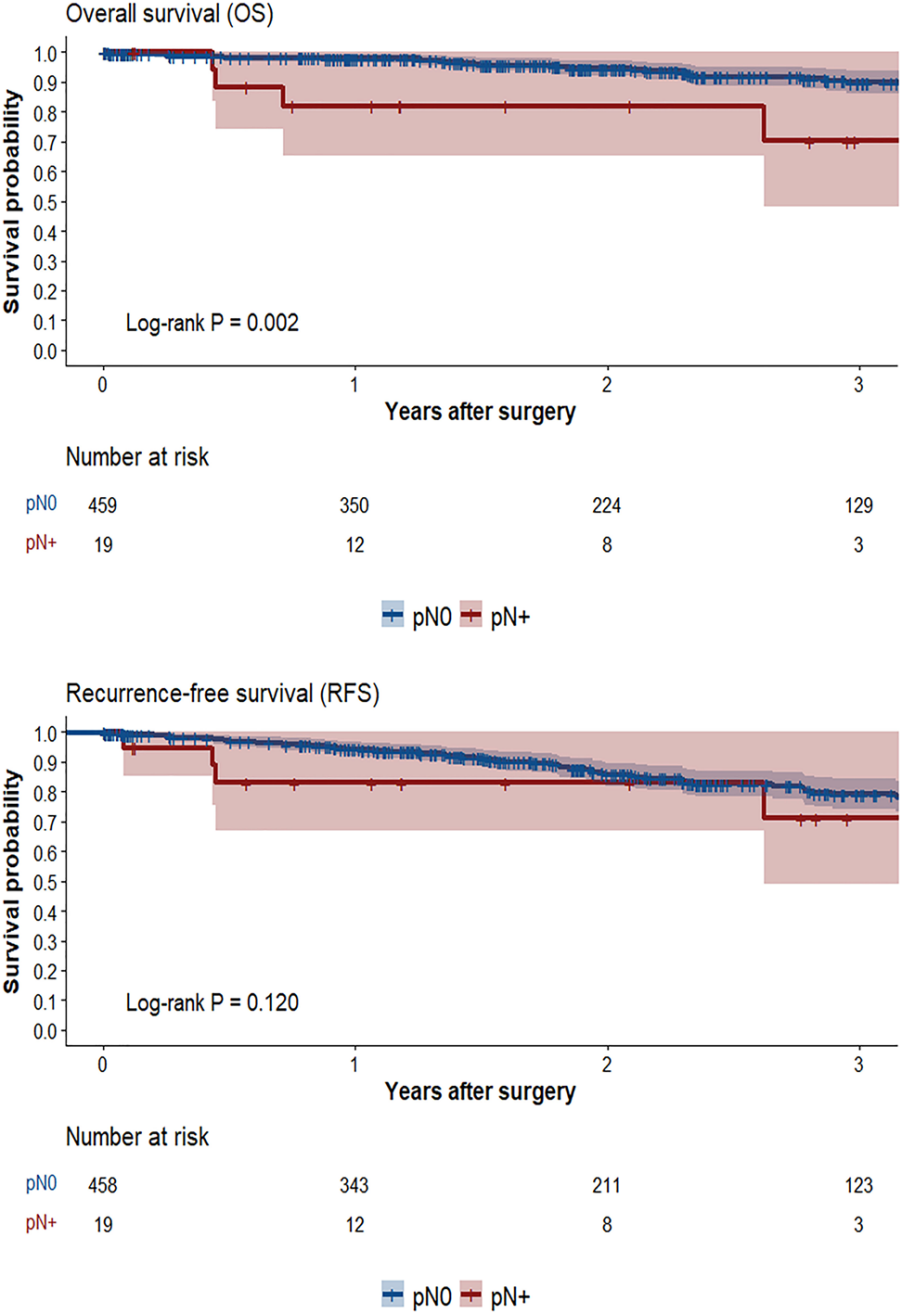
Overall survival and recurrence-free survival between pN0 and pN + subgroups

Over a median follow-up period of 23.5 months (IQR 12.6–39.0), there were no significant differences in locoregional recurrence (5.3% vs. 5.4%, *P* = 1.000) and distant recurrence (10.5% vs. 4.6%, *P* = 0.231) or RFS (*P* = 0.120, 71.1% [95% confidence interval (95% CI 49.1–100.0%)] vs. 79.0% (95% CI 74.1–84.2%) at 3 years) between patients with and without pN+. However, OS was lower in patients with pN + compared to those with pN0 (*P* = 0.002, 70.2% (95% CI 48.1–100.0%) vs. 89.7% (95% CI 85.9–93.6%) at 3 years). (Fig. 1; Table 3).

### Subgroup analysis

In the subset of patients with a SUVmax ≥ 4.5, pN + was identified in 7.3% (12/163) of cases, correlating with a lower OS compared to pN0 (*P* = 0.002, 58.2% (95% CI 33.0–100.0%) vs. 85.8% (95% CI 78.6–93.6%) at 3 years). For patients with a SUVmax < 4.5, the incidence of pN + was 2.2% (7/315), with no significant difference in OS observed between pN + and pN0 patients. In both subsets, no significant differences in recurrence rates and RFS were found between the two groups (Supplementary Fig. 1; Supplementary Table 2).

## Discussion

This multicenter retrospective cohort study identified a 4.0% incidence of pN + (2.1% pN1, 1.3% pN2, and 0.6% pN1 + 2) procedure specifically in patients with cIA1-2 NSCLC following segmentectomy without frozen section. This indicates that unforeseen pN + occurs very rarely in this group of patients. Consistently, a recent study from Nobel TB and colleagues [8] focusing on segmentectomies for clinical stage IA NSCLC identified similar rates of nodal upstaging as this study, with an overall rate of 4.0% and specific nodal upstaging rates of 3% for pN1 and 1% for pN2. Additionally, the unexpected pN + rate in this study is lower than the rates reported in the JCOG 0804 (6.2%) [4] and the German DRKS 00004897 trials (5.7%) [5], where a frozen section was performed on suspicious lymph nodes, and lobectomy was carried out if the lymph nodes were found to be positive. These discrepancies between our findings and theirs may originate from the proportion of solid tumors.

The notable observation from our study was that SUVmax can predict pN+, and pN + occurred more frequently in higher F-fluorodeoxyglucose avidity tumors. Consistently, a recent review highlighted the predictive accuracy of SUVmax in identifying occult nodal metastasis, with findings indicating an accuracy range of 55–77% [9]. Furthermore, Li et al. [10] found that a SUVmax cut-off of 4.3 predicted occult nodal metastasis in clinical stage I NSCLC. Tapias et al. [11] reported that SUVmax values of ≥ 2.9 in adenocarcinoma and ≥ 7.2 in squamous cell carcinoma correlated with increased rates of nodal upstaging. Besides preoperative radiologic markers, circulating tumor DNA may be useful for predicting pN + in terms of molecular markers [12]. Moreover, the histologic component should be considered as a potential additional factor. Indeed, we found that the highest likelihood of nodal upstaging was in the adenocarcinoma cohort (84.2%), which aligns with previous results [8]. Thus, optimizing preoperative assessments is valuable for thoracic surgeons in developing intraoperative lymph node assessment strategies. Intraoperatively applying indocyanine green and rapid immunohistochemistry techniques may potentially help to identify pN + [13, 14]. Because performing a frozen section only on the hilar nodes may not be thorough enough to identify all cases of pN+, as our results suggest.

Compared to locoregional recurrence rates after segmentectomy in the JCOG 0802 (11.2%) [4] and CALGB 140503 trial (12.3%, frozen section on hilar lymph nodes during segmentectomy was mandatory before randomization) [15], our findings showed locoregional recurrence rates in both the overall cohort and subsets to be nearly half of those reported. Despite that there were no completion lobectomies performed for cases with pN + in our study, no increase in locoregional recurrence rates was detected when compared to cases with pN0, similar to the findings in the study from Nobel et al. [8]. In addition, the distant recurrence rate was not significantly influenced by pN+, possibly because most patients with pN + received systemic adjuvant chemotherapy. Distant recurrence is often considered a systemic disease related to complex biologic interactions. The corresponding comparable outcomes were observed when we analyzed recurrence as a time to event using RFS. Interestingly, our 3-year RFS in pN0 was higher than in the CALGB 140503 trial [15] but lower than in the JCOG 0802 trial [4]. The reasons for this could be further analyzed in future research with larger, more diverse populations.

Our study showed that patients with unforeseen pN + had inferior OS compared to those with pN0, which is not surprising. The 3-year OS was similar to the CALGB 140503 trials [15] for pN0 patients but lower than in the JCOG 0802 trial [4]. Additionally, in the subgroup analysis, we verified that cases with pN + exhibited poorer survival rates compared to those with pN0 in the hypermetabolic group, but not in the hypometabolic group. This might further suggest that the importance of preoperative PET examination for the intraoperative assessment for lymph nodes.

Last but not least, recent trials have shown the efficacy of targeted treatments for tumors with actionable genetic alterations and immunotherapy in the adjuvant setting [16–19]. These developments suggest enhanced therapeutic options for clinical early-stage NSCLC with unforeseen lymph node involvement, offering improved treatment possibilities and justifying a more aggressive preoperative lymph node staging in patients with hypermetabolic primary tumors on PET scans.

This study has several limitations. Firstly, the retrospective nature of using a multicenter database may introduce selection bias. Furthermore, the sample size for the objective event seems small, limiting further analysis, such as examining differences between N1 and N2 involvement, and potentially lacking the power to robustly support the conclusion. Moreover, specific data such as adenocarcinoma subtypes were not available due to the original study design, further restricting our ability to perform detailed analyses. As highlighted in this study, the SUVmax value is closely linked to cancer metabolism and biologic characteristics. While it is well known that solid or micropapillary adenocarcinoma subtypes tend to be more aggressive, we were unfortunately unable to evaluate the impact of different adenocarcinoma patterns on SUVmax values. Secondly, relying on statistical imputation for missing data could lead to reporting bias. Thirdly, regarding intraoperative lymphadenectomy, a detailed audit of procedural quality was not conducted in this study, introducing possible confounding variables that may affect the examination of pN+. However, the optimal approach to lymph node dissection, whether systematic or selective, remains controversial.

## Conclusion

Unforeseen nodal upstaging after segmentectomy for cIA1-2 NSCLC is rare, particularly in cases with low metabolic activity in the primary tumor. However, in patients with high F-fluorodeoxyglucose-avidity of the primary tumor the rate of nodal upstaging was more than three-fold higher in our series. This warrants more aggressive preoperative and intraoperative nodal assessment to appropriately inform treatment decision.

## Supplementary Information

Below is the link to the electronic supplementary material.

Supplementary file1 (DOCX 681 KB)
