# Correction: Unforeseen nodal upstaging in patients undergoing segmentectomy without frozen section: a multicenter retrospective cohort study

**DOI:** 10.1007/s00464-025-11668-7

**Published:** 2025-03-14

**Authors:** Lin Huang, Alessandro Brunelli, Demetrios Stefanou, Edoardo Zanfrini, Abid Donlagic, Michel Gonzalez, René Horsleben Petersen

**Affiliations:** 1 Department of Cardiothoracic Surgery, Copenhagen University Hospital, Rigshospitalet, Blegdamsvej 9, 2100 Copenhagen Ø, Denmark; 2 Department of Thoracic Surgery, St. James’s University Hospital, Leeds, UK; 3 Department of Thoracic Surgery, University Hospital of Lausanne, Lausanne, Switzerland

**Surgical Endoscopy** 10.1007/s00464-025-11612-9

The original online version of this article was revised to correct the presentation of the name of coauthor Demetrios Stefanou, and to add ORCID information for coauthors Lin Huang, Alessandro Brunelli, and Michel Gonzalez.

The original article has been corrected.

