# Supplementary material for: Unforeseen nodal upstaging in patients undergoing segmentectomy without frozen section: a multicenter retrospective cohort study
Source: Surg Endosc. 2025 Feb 13;39(4):2296–303. doi: 10.1007/s00464-025-11612-9 (PMC11933133; doi:10.1007/s00464-025-11612-9)

| **Supplementary Table 1. The pathological nodal status after segmentectomy for clinical stage IA1-2 in 3 centers participating in the study.** | | | | |
| --- | --- | --- | --- | --- |
| **Pathological nodal status^a^** | **Total**  **(n = 478)** | **Center 1**  **(n = 93)** | **Center 2**  **(n = 217)** | **Center 3**  **(n = 168)** |
| pN0 | 459 (96.0%) | 92 (98.9%) | 208 (95.9%) | 159 (94.6%) |
| pN1 | 10 (2.1%) | 1 (1.1%) | 5 (2.3%) | 4 (2.4%) |
| pN2 | 6 (1.3%) | 0 (0.0%) | 2 (0.9%) | 4 (2.4%) |
| pN1+2 | 3 (0.6%) | 0 (0.0%) | 2 (0.9%) | 1 (0.6%) |
| a: it was presented as number (proportion). | | | | |

| **Supplementary Table 2. Recurrence between pN0 and pN+ in subgroups of maximum standardized uptake values (SUVmax) in positron emission tomography scan ≥ 4.5 and < 4.5.** | | | | | | |
| --- | --- | --- | --- | --- | --- | --- |
|  | **Any recurrence** | **P-value** | **Locoregional recurrence** | **P-value** | **Distant recurrence** | **P-value** |
| **SUVmax < 4.5 (n = 315)** |  | 1.000 |  | 1.000 |  | 1.000 |
| pN0 (n = 308) | 21 (6.8%) |  | 12 (3.9%) |  | 9 (2.9%) |  |
| pN+ (n = 7) | 0 (0.0%) |  | 0 (0.0%) |  | 0 (0.0%) |  |
| **SUVmax** ≥ **4.5 (n = 163)** |  | .435 |  | .974 |  | .299 |
| pN0 (n = 151) | 25 (16.6%) |  | 13 (8.6%) |  | 12 (7.9%) |  |
| pN+ (n = 12) | 3 (25.0%) |  | 1 (8.3%) |  | 2 (16.7%) |  |

**Supplementary Figure 1. Overall survival and recurrence-free survival between pN0 and pN+ in subgroups of maximum standardized uptake values (SUVmax) in positron emission tomography scan ≥ 4.5 and < 4.5.**


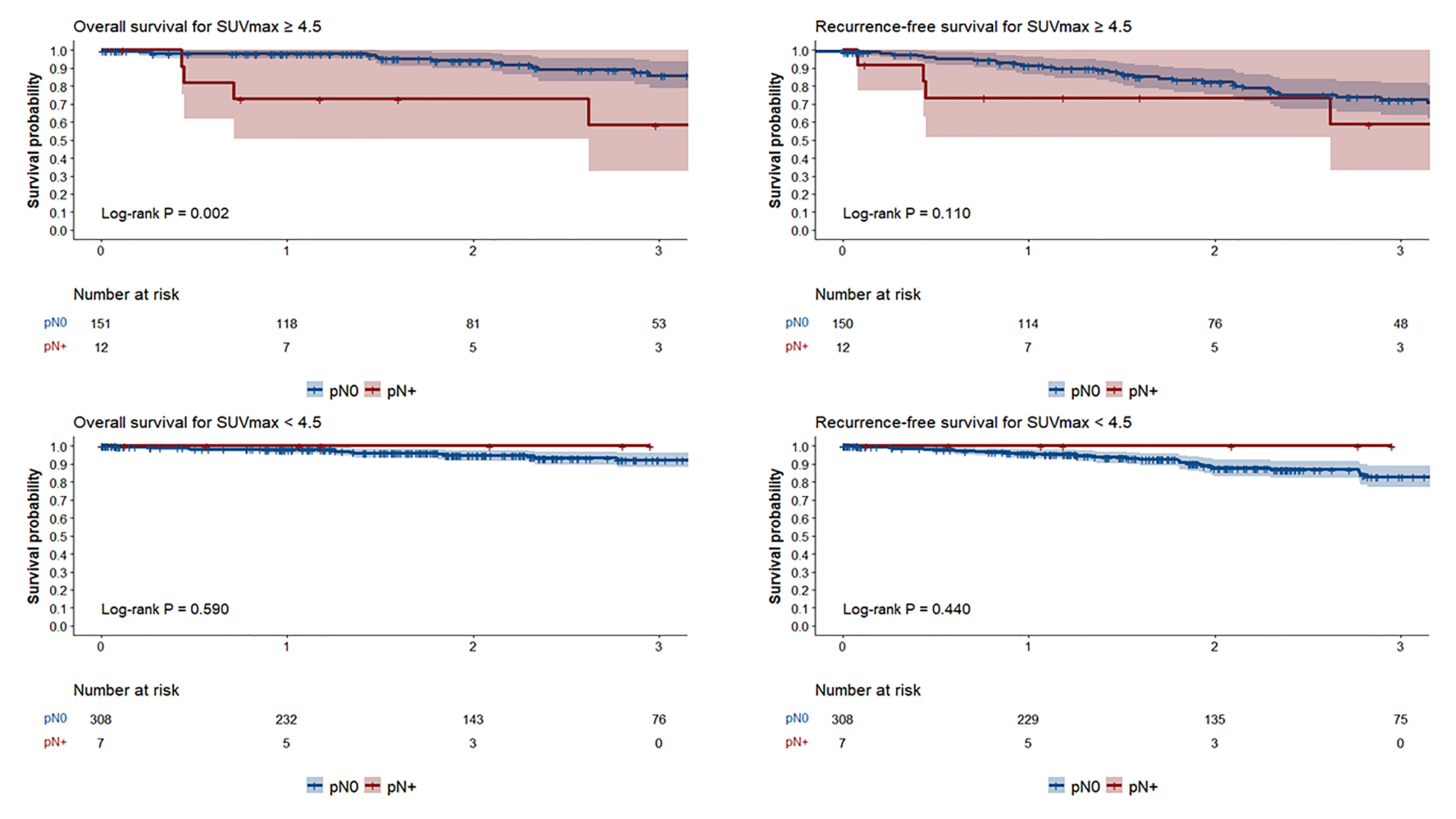

Supplement: Supplementary file 1 — Supplementary file1 (DOCX 681 KB) [file 464_2025_11612_MOESM1_ESM.docx]
